# Isolated Ocular Relapse of Acute Myeloid Leukaemia Post Allogeneic Stem Cell Transplant

**DOI:** 10.1155/2024/2235819

**Published:** 2024-02-21

**Authors:** M. H. Tong, A. Kwok, A. Walsh, P. Heydon, E. S. Koh, N. McNamara, A. Bryant

**Affiliations:** ^1^Department of Haematology, Liverpool Hospital, Liverpool, New South Wales, Australia; ^2^Department of Ophthalmology, Liverpool Hospital, Liverpool, New South Wales, Australia; ^3^Department of Radiation Oncology, Liverpool Hospital, Liverpool, New South Wales, Australia; ^4^South West Sydney Clinical School, University of New South Wales, Sydney, New South Wales, Australia

## Abstract

We present a rare case of a 39-year-old female with extramedullary relapse of acute myeloid leukaemia (AML) isolated to the left eye 2 months post allogeneic haematopoietic stem cell transplant. She initially presented with painless left eye erythema, swelling, and visual impairment. Initial ophthalmology review revealed conjunctival chemosis, raised intraocular pressure, and serous retinal detachments. She was initially treated for suspected orbital cellulitis with intravenous antibiotic and antifungal therapy but clinically progressed so was then treated with intravenous corticosteroids. One week later, she progressed to angle-closure glaucoma with development of a hypopyon and an enlarging subconjunctival mass. She proceeded to urgent subconjunctival biopsy and drainage of subretinal fluid which confirmed extramedullary relapse of AML. Notably, further investigation found no evidence of bone marrow or central nervous system relapse. She proceeded to localized radiotherapy with gradual resolution of the subconjunctival mass and serous retinal detachment and was for consideration of donor lymphocyte infusions and azacitidine therapy; unfortunately, she developed respiratory sepsis and passed away despite maximal efforts. This case represents a rare and unusual presentation of isolated ocular extramedullary relapse of AML and emphasises the importance of early ophthalmology involvement and tissue biopsy when there is high clinical suspicion of the disease.

## 1. Introduction

The key treatment with curative intent in high-risk acute myeloid leukaemia (AML) is allogeneic haematopoietic stem cell transplantation. Unfortunately, there remains the persistent risk of relapse after transplant. The most common sites of relapse apart from the bone marrow are the skin, gastrointestinal system and central nervous system (CNS). Ocular relapse, in contrast, is a more uncommon site of relapse that is most often seen alongside aggressive CNS and bone marrow relapse [1, 2]. Ophthalmic manifestations can occur from direct invasion of leukaemic cells and indirect effects from malignancy or from local or systemic effects of treatment. We report a case of isolated ocular relapse of acute myeloid leukaemia which interestingly showed no evidence of bone marrow or CNS involvement at the time of diagnosis.

## 2. Case Study

A 39*-*year-old female was initially diagnosed with AML after a core biopsy of a breast lump showed myeloid sarcoma with a subsequent bone marrow biopsy in December 2019 confirming AML with complex karyotype. She proceeded to 7-3 induction chemotherapy followed by 1 cycle of high-dose cytarabine consolidation. Repeat bone marrow biopsy confirmed morphological remission, and she proceeded to a busulfan-cyclophosphamide conditioning sibling-matched allogeneic haematopoietic stem cell transplant on the 11^th^ of March 2020. Her initial post-transplant recovery period was unremarkable apart from a mild skin rash from day 9 post-transplant which was treated as graft versus host disease with cyclosporine. This was gradually weaned with no progression of skin rash and no other manifestations of graft vs. host disease.

**Figure 1:**
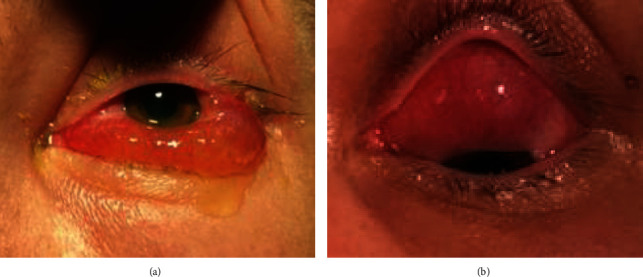
(a) Left conjunctival chemosis. (b) Solid subconjunctival superior bulbar mass.

On day 57 after her transplant, she presented with painless left eye redness and swelling. On examination of the left eye, there was mild visual impairment to 6/9, mild periorbital oedema with significant conjunctival chemosis causing lag ophthalmos and limited extraocular motility, raised intraocular pressure to 38 mmHg, and anisocoria with an unresponsive, dilated pupil. Her left anterior chamber demonstrated 1+ white cell activity, and the posterior segment had serous retinal detachments without any evidence of optic disc swelling, retinitis, or vasculitis. She was admitted to hospital and commenced on systemic antifungals and antibiotics for the suspected differentials of orbital cellulitis and infectious scleritis alongside topical steroid drops and glaucoma treatment. CT imaging showed subtle left lacrimal gland thickening, but biopsy of the lacrimal gland showed nonspecific inflammatory changes with no evidence of AML or post-transplant lymphoproliferative disease. Due to progression to extensive enlarging serous retinal detachments, she received three pulsed doses of 1 gram intravenous methylprednisolone. This led to resolution of her severe conjunctival chemosis. With resolution of the chemosis, a solid subconjunctival mass was noted underlying the superior bulbar conjunctiva adherent to the globe (Figure 1). She was discharged with close ongoing outpatient follow-up with weaning oral prednisolone, topical steroid drops, and topical glaucoma treatment.

**Figure 2:**
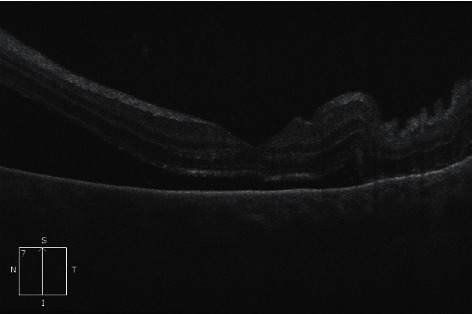
Vertical OCT raster of the macula demonstrating retinal folds and dependent subretinal fluid from exudative retinal detachment.

**Figure 3:**
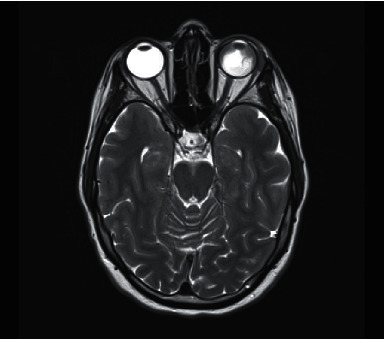
Exudative retinal detachments and thickening of globe tissue seen on MRI orbits (T2 sequence).

Our patient represented 1 week after discharge with left eye pain, headache, nausea, and vomiting. On examination of her left eye, her vision had deteriorated to counting fingers, her intraocular pressure was 55 mmHg, a hypopyon was present, and her anterior chamber angle was closed at 270 degrees with anterior rotation of the ciliary body visible superiorly. Vertical optical coherence tomography (OCT) raster of the macula demonstrated retinal folds and dependent subretinal fluid from exudative retinal detachment (Figure 2). 45-degree fluorescein angiography was performed and demonstrated diffuse pooling of due within the exudative retinal detachments. There was no retinal vascular leak or ischaemia suggestive of leukaemic stasis retinopathy. The quality of the images was limited by conjunctival chemosis and the peripheral anatomical position of the pathology. She was diagnosed with secondary acute angle-closure glaucoma due to rapidly enlarging serous retinal detachments. A FDG-PET scan showed moderate to intense metabolically active soft tissue thickening in the left globe, and an MRI of the brain showed exudative retinal detachments and thickening of the globe tissue (Figure 3).

**Figure 4:**
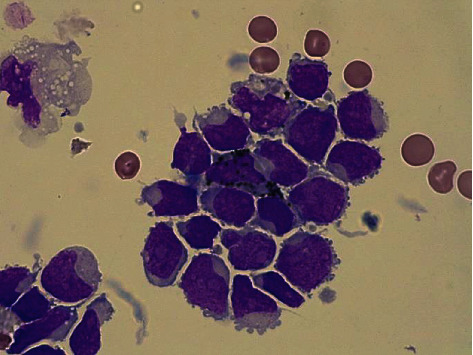
Infiltrate of blast cells seen on cytospin from scleral biopsy. *Photo taken on 40x high-power magnification.*

**Figure 5:**
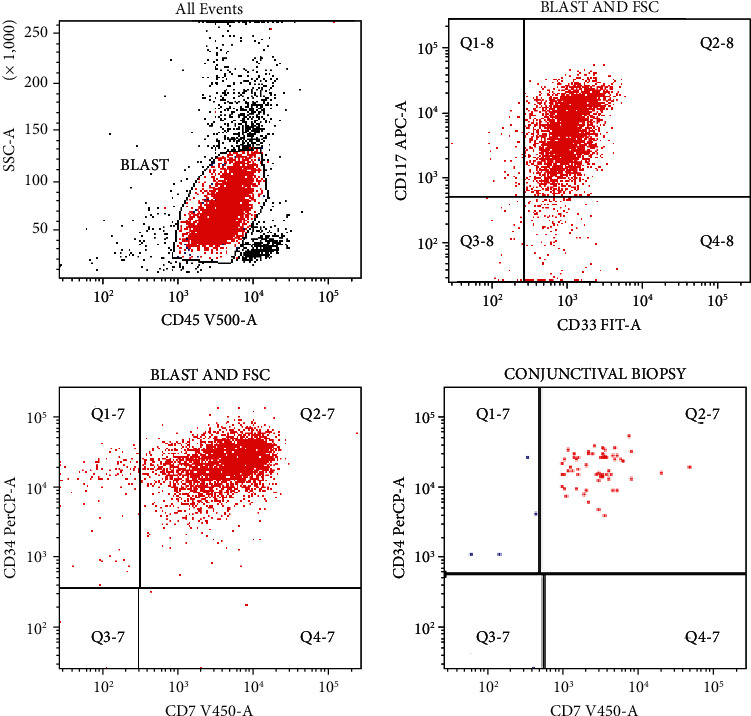
Flow cytometry from subconjunctival biopsy, superior sclerotomy, left eye. The first 3 flow plots show the population of blast cells with CD34 (surface marker indicative of blast cells) with an aberrant CD7. This is the original population seen on the initial diagnosis of AML for the patient. The conjunctival biopsy demonstrated the same population with CD34- and CD7-positive blast cells.

A further two pulsed doses of 1 gram intravenous methylprednisolone was given alongside maximal medical glaucoma treatment followed by urgent biopsy of the subconjunctival mass and drainage of the subretinal fluid via an ab-externo approach with subsequent improvement of secondary angle closure. Cytospin from this subretinal fluid showed a significant blast cell infiltrate (Figure 4), and flow cytometry of the subconjunctival biopsy confirmed a prominent CD7+ and CD34+ monomorphic population which was the same myeloid population seen on her initial diagnosis (Figure 5), confirming the diagnosis of ocular relapsed AML. Samples were also sent for histopathology and similarly demonstrated haematopoietic blast cells consistent with AML. The combination of a blast infiltrate within the scleral and subretinal fluid biopsies was consistent with an exudative retinal detachment, rather than alternatives such as steroid-related central serous chorioretinopathy.

Rather surprisingly, subsequent bone marrow biopsy and lumbar puncture did not demonstrate any evidence of relapse, and there was 100% chimerism on her bone marrow molecular studies. Thus, the diagnosis was confirmed as extramedullary relapse of AML limited to the left globe. Immunosuppressive therapy was rapidly weaned, and she proceeded to receive 20 Gy in 5 daily fractions of palliative radiotherapy to the left eye with improvement of her visual symptoms and gradual resolution of the subconjunctival mass and serous retinal detachments. She was scheduled to commence azacitidine therapy alongside donor lymphocyte infusions.

Unfortunately, 7 weeks after completing radiotherapy, she presented with cough, shortness of breath, and fevers. Subsequent imaging showed bilateral lung infiltrates. Despite prompt commencement of antibiotics, antifungals, and antiviral therapy as broad cover for respiratory sepsis, she continued to deteriorate culminating in developing type 1 respiratory failure requiring intubation and ventilation. She was commenced on methylprednisolone to cover for potential leukaemic lung infiltrates as she was too unstable to consider a lung biopsy to clarify the cause of these infiltrates. Despite ongoing maximal support, she continued to deteriorate and sadly passed away.

## 3. Discussion

This case is a rare presentation of extramedullary ocular relapse of AML soon after haematopoietic allogeneic stem cell transplantation without associated CNS or bone marrow involvement. The 5-year cumulative incidence of isolated extramedullary relapse of AML after allogeneic transplant has recently reported to be 4%, with poor cytogenetics and prior extramedullary disease being independent risk factors predicting extramedullary relapse [3]. The most common sites of relapse are where leukaemic cells can evade the immune surveillance driven by graft versus leukaemia effect after transplant [4]. These sites include the CNS, gastrointestinal tract, breast, skin, testes, and ovaries [2, 4]. Furthermore, in some cases, bone marrow can remain in clinical remission at time of extramedullary relapse [2, 5].

There are very few reported cases of isolated ocular extramedullary relapse of AML without CNS or bone marrow involvement. As red eye is a very common presenting complaint, there is often a lag in definitive diagnosis as other processes including infection and post-transplant lymphoproliferative disease need to be excluded. The ophthalmic differentials for conjunctival chemosis and vision loss are broad in post-transplant patients. Microbial orbital cellulitis, particularly in association with invasive fungal sinusitis, is an important life-threatening differential, particularly as its likelihood increases in cytopaenic patients [6]. Post-transplant lymphoproliferative disorder most commonly occurs in the first 2 to 6 months following allogeneic stem cell transplantation [7]. Graft versus host disease inflammatory scleritis has also been rarely reported [8, 9].

In our case, given the anterior chamber activity with hypopyon and serous retinal detachments, there was high suspicion for leukaemic relapse. CT imaging initially demonstrated left lacrimal gland thickening which prompted biopsy, but the results were noncontributory. Subsequent MRI imaging showed circumferential soft tissue thickening of the globe, and it was only a second biopsy of the subconjunctival mass that was revealed with resolution of chemosis alongside cytospin of the subretinal fluid, which confirmed AML relapse. In retrospect, given the subsequent serial progress imaging, on multidisciplinary imaging review, it was thought that the initial globe thickening likely reflected relapsed disease.

The prevalence of ocular leukaemic involvement has a wide range reported between 50 and 80% with close correlation with CNS and bone marrow involvement [1, 10]. However, isolated ocular relapse without systemic involvement of AML is particularly rare. To our knowledge, there are 3 reported cases of this in the paediatric population with ocular presentations of hypopyon [11] and choroidal infiltrates with serous retinal detachment [12, 13], while the 5 reported adult cases had ocular presentations of choroidal infiltrates [1, 14, 15], conjunctival infiltrates [10], and hypopyon [16]. Two of the reported paediatric cases presented with choroidal infiltrates culminating in serous retinal detachments [12, 13]. Our case, in contrast, is an adult presentation of serous retinal detachment with secondary angle closure and no retinopathy which is much rarer and generally presents as active disease in acute lymphoblastic leukaemia as opposed to AML. Furthermore, the usual presentation would be of a posterior pole shallow retinal detachment [12] which was also not seen in our patient.

Isolated extramedullary relapse of AML often precedes eventual systemic relapse, including bone marrow involvement [1, 4, 12, 13, 15, 17, 18]. However, there have also been cases reported where patients remained in bone marrow remission despite extramedullary relapse [3]. Regardless, prognosis is quite poor in the setting of extramedullary relapse post stem cell transplant with 3-year survival after isolated extramedullary relapse being 30.1% [3]. Due to the high risk of progression to systemic relapse, most cases have used combination therapy including radiotherapy, systemic chemotherapy, and donor lymphocyte infusion with variable outcomes [2, 4, 5, 19]. Currently, there is no consensus or guidelines regarding optimal treatment, and the choice of treatment varies depending on age, comorbidities, and disease burden at the time of relapse.

## 4. Conclusion

Isolated ocular relapse of AML without bone marrow or CNS involvement is a rare presentation, and diagnosis is often delayed due to the nonspecific presentation with red eye, pain, and swelling. Ophthalmology must be involved early to aid in diagnosis and management. As seen in this case, given the high suspicion of relapse, it is important to expedite tissue biopsy to confirm diagnosis. Ocular relapse renders a high risk of eventual systemic relapse; hence, treatment should involve a combination of both local and systemic therapies.
